# 16S rRNA amplicon sequencing identifies microbiota associated with oral cancer, human papilloma virus infection and surgical treatment

**DOI:** 10.18632/oncotarget.9710

**Published:** 2016-05-30

**Authors:** Rafael Guerrero-Preston, Filipa Godoy-Vitorino, Anne Jedlicka, Arnold Rodríguez-Hilario, Herminio González, Jessica Bondy, Fahcina Lawson, Oluwasina Folawiyo, Christina Michailidi, Amanda Dziedzic, Rajagowthamee Thangavel, Tal Hadar, Maartje G. Noordhuis, William Westra, Wayne Koch, David Sidransky

**Affiliations:** ^1^ Department of Otolaryngology and Head and Neck Surgery, Johns Hopkins University School of Medicine, Baltimore, Maryland, USA; ^2^ Department of Obstetrics and Gynecology, University of Puerto Rico School of Medicine, San Juan, Puerto Rico; ^3^ Natural Sciences Department, Microbial Ecology and Genomics Laboratory, Inter American University of Puerto Rico, Metropolitan Campus, San Juan, Puerto Rico; ^4^ Department of Molecular Microbiology and Immunology, Johns Hopkins University School of Public Health, Baltimore, Maryland, USA; ^5^ Department of Microbiology, Icahn School of Medicine at Mount Sinai, New York, New York, USA; ^6^ Department of Otorhinolaryngology-Head and Neck Surgery, University of Groningen, University Medical Center, Groningen, The Netherlands; ^7^ Department of Pathology-Surgical Pathology, Johns Hopkins University School of Medicine, Baltimore, Maryland, USA

**Keywords:** microbiome, 16s rRNA, oral cancer, oropharyngeal cancer, human papilloma virus (HPV)

## Abstract

Systemic inflammatory events and localized disease, mediated by the microbiome, may be measured in saliva as head and neck squamous cell carcinoma (HNSCC) diagnostic and prognostic biomonitors. We used a 16S rRNA V3-V5 marker gene approach to compare the saliva microbiome in DNA isolated from Oropharyngeal (OPSCC), Oral Cavity Squamous Cell Carcinoma (OCSCC) patients and normal epithelium controls, to characterize the HNSCC saliva microbiota and examine their abundance before and after surgical resection.

The analyses identified a predominance of Firmicutes, Proteobacteria and Bacteroidetes, with less frequent presence of Actinobacteria and Fusobacteria before surgery. At lower taxonomic levels, the most abundant genera were *Streptococcus, Prevotella, Haemophilus, Lactobacillus* and *Veillonella*, with lower numbers of *Citrobacter* and Neisseraceae genus *Kingella*. HNSCC patients had a significant loss in richness and diversity of microbiota species (p<0.05) compared to the controls. Overall, the Operational Taxonomic Units network shows that the relative abundance of OTU's within genus *Streptococcus, Dialister*, and *Veillonella* can be used to discriminate tumor from control samples (p<0.05). Tumor samples lost *Neisseria*, *Aggregatibacter* (Proteobacteria), *Haemophillus* (Firmicutes) and *Leptotrichia* (Fusobacteria). Paired taxa within family Enterobacteriaceae, together with genus *Oribacterium*, distinguish OCSCC samples from OPSCC and normal samples (p<0.05). Similarly, only HPV positive samples have an abundance of genus *Gemellaceae* and *Leuconostoc* (p<0.05). Longitudinal analyses of samples taken before and after surgery, revealed a reduction in the alpha diversity measure after surgery, together with an increase of this measure in patients that recurred (p<0.05). These results suggest that microbiota may be used as HNSCC diagnostic and prognostic biomonitors.

## INTRODUCTION

Preliminary studies suggest that microbiota markers in saliva may be useful diagnostic and prognostic biomonitors for diverse health conditions [1–3]. Both systemic inflammatory events and localized disease, such as periodontal disease [4, 5] and head and neck squamous cell carcinoma (HNSCC) [6], may be mediated by the oral microbiome.

There were an estimated 300,400 newly diagnosed cases and 145,000 deaths from oral cavity cancer (including lip cancer) in 2012 worldwide. There were an estimated 86,700 new cases of nasopharyngeal carcinoma and 50,800 deaths in 2012 [7, 8]. A majority of HNSCC patients are diagnosed with tumors of the oral cavity (OSCC) and the oropharynx (OPSCC). It is estimated that, in 2015 alone, there will be approximately 45,780 new cases of cancer of the oral cavity and larynx and 8,650 estimated deaths attributable to these cancers in the United States [8]. The major known etiological factors for oral and oropharyngeal cancer are smoking, alcohol consumption and high-risk human papilloma virus (HPV) infection. It is also known that the distribution and occurrence of oral cancer cases varies by age, ethnic group, culture, life-style, and level of country development [9]. For example, the Population Attributable Risk (PAR) due to the effects of tobacco and alcohol on oral cavity cancers is lower in the United States [10] than in Europe and Latin America [10]. However, the etiological role of bacteria in HNSCC has not been previously examined in detail [11].

The role of bacteria in the development of HNSCC cancer has not been delineated, but the persistent presence of bacteria at tumor sites in the oral cavity and oropharynx raises intriguing questions about the role of bacteria in the progression and treatment of HNSCC. Unfortunately, to date some studies have included only cultured oral bacterial species, using classical cloning and sequencing approaches [12, 13]. More recent studies of the oral microbiota have examined patients with OSCC and precancerous lesions, but they have been limited in scope and provide inconsistent results [14–16].

High-throughput technology for understanding the ecology of microbial ecosystems is increasingly being used to test hypotheses and build experimental strategies aimed at revealing the role of bacteria in health and disease [17]. The method of excellence for performing this characterization is the use of small-subunit ribosomal RNA (16S rRNA) studies, which bypasses the need for culturing. Instead, the 16S rRNA gene sequences (for archaea and bacteria) are used as stable phylogenetic markers to identify taxonomic lineages in a given sample [18, 19]. The 16S rRNA gene has nine variable regions: a combination of variable and moderately conserved regions is optimal for performing analyses at different phylogenetic depths, and the V3-V5 region is one of the preferred regions for characterizing the communities with few errors for taxonomy assignment [20–22].

Recent approaches using 454 parallel sequencing of the 16S rRNA were used to assess the diversity and relative abundance of bacteria in the saliva of OSCC patients and revealed a majority of Firmicutes and Bacteroidetes with only fifteen unique OTUs associated to OSCC patients [16, 19]. The oral microbiome has previously been related to the establishment and progression of precancerous lesions and neoplasms in the oral cavity [23, 24]. In oral cancer samples from both a discovery and a validation cohort, abundance of *Firmicutes* (especially *Streptococcus*) and *Actinobacteria* (especially *Rothia*) was significantly decreased relative to anatomically matched contralateral normal samples after sequencing 16S rRNA hypervariable V4 region amplicons [14].

The significance of these cross-sectional findings is not yet clear. Furthermore, the microbial diversity and taxonomic composition of human oral microbiota associated with HPV positive and HPV negative tumors of the head and neck have not been examined and there have not yet been any attempts to examine the longitudinal associations between the microbiota in patients with cancer of the oral cavity and the oropharynx. Similarly, there have not been any longitudinal reports of the association between the pre- and post-treatment saliva microbiome of HNSCC.

We hypothesized that the microbiota provides a broad spectrum of taxa identification, a distinct sequence-read record, and robust detection sensitivity, which can be used to develop saliva-based diagnostic tests of HPV positive and HPV negative, oral cavity and oropharyngeal cancer. In the present study, we did a cross-sectional comparison of the microbial communities present in saliva DNA from HPV positive and HPV negative patients with cancer of the oropharynx, cancer of the oral cavity, and normal oral cavity epithelium. We selected between 1-4 additional saliva samples collected in subsequent visits, from 10 of the 19 HNSCC patients to evaluate the longitudinal association between the microbiota and treatment effects.

## RESULTS

### Study patients

This study was nested within a longitudinal cohort study of 787 patients who presented with histopathologically confirmed HSNCC (this includes patients who presented for treatment of a recurrence after primary treatment at an outside hospital) to the outpatient clinic of the Johns Hopkins Hospital in Baltimore, Maryland from 2000 to 2011. Patients were included if they had at least one post-treatment salivary sample and consented for the study. All patients had undergone treatment with curative intent. The study protocol was approved by the institutional review board of the Johns Hopkins Hospital, as well as by the Johns Hopkins Institutional Review Board. Written informed consent was obtained from all patients.

Patients were consented for this study under the molecular surveillance clinical research protocol. Saliva was collected from 44 patients: 25 patients with no history of cancer and 19 HNSCC patients. Longitudinal saliva samples were collected from 58% of the HNSCC patients, totaling 62 samples. Nonetheless, we eliminated 3 samples from 2 patients (2 OSCC and 1 OPSCC), 2 of them with unknown HPV status, as well as the only HPV positive OSCC sample. The analyses presented here are based on a total of 59 saliva samples acquired from 42 patients; of these, 34 saliva samples corresponded to 17 HNSCC patients and 25 saliva samples corresponded to 25 controls without cancer, which also had negative smoking and drinking histories. Most of the HNSCC patients were OPSCC patients (7 were HPV positive and 4 were HPV negative), and the rest were OPSCC patients (all 6 were HPV negative) (Table 1).

**Table 1:** Patients, number of samples, total number of sequences and Operational Taxonomic Units by histology type and HPV status

Sample type (Histology+HPV status)	Number of Patients	Number of Samples	Total Number of sequences	Total Number of OTUs
Normal Mucosa (Control) HPV Negative	25	25	197,743	13,849
Oropharynx Squamous cell carcinoma (OPSCC) HPV Negative	4	11	88,488	2,326
Oropharynx Squamous cell carcinoma (OPSCC) HPV Positive	7	13	125,608	2,924
Oral Cavity Squamous cell carcinoma (OSCC) HPV Negative	6	10	78,330	3,659
**Grand Total**	42	59	496,017	23,734

Tissue and saliva samples were collected between 2000 and 2011 and stored in the Johns Hopkins Head and Neck Cancer Research Division's Tumor Bank, from where they were randomly selected for this study (Supplementary Table 1). Fifty-eight percent (58%) of the HNSCC patients chosen for the study had OPSCC. There was no difference in median age (66 and 62 years) or percentage of male patients (67% and 77%) between OSCC and OPSCC patients. Forty-seven percent (47%) of the HNSCC patients were HPV positive. Most HNSCC patients were former smokers or never smokers: OPSCC (72%) and OSCC (63%). Similarly, most HNSCC patients were occasional drinkers or non-drinkers: OPSCC (72%) and OSCC (63%). Approximately half of the patients were diagnosed with T1 or T2 stage tumors: OSCC (50%) and OPSCC (55%). Most patients had nodal involvement: OSCC (75%) and OPSCC (73%). None of the patients had known metastasis. All the OPSCC were treated with surgery and chemoradiation, compared to only 25% of the OSCC. Most of the OSCC patients (63%) only required surgical treatment (Supplementary Table 2).

We acquired a set of 607,646 raw reads when we sequenced the 16S rRNA V3–V5 hypervariable region from the 62 DNA samples used in the study. The number of sequences per sample ranged from 3,857 to 21,379 and had an average of 9,645 sequences per sample (Supplementary Table 3). Sequences underwent strict quality and size filtering, removing reads shorter than 150bp, as well as those with mismatches and poor quality scores. Sequences were then error-corrected using the Acacia tool, followed by de novo chimera detection with the UCHIME program, and screening for chloroplast contaminant sequences. In total, 490,169 sequences passed preprocessing. The 490,169 sequences were binned into 8,771 OTUs. Control samples had the highest number of sequences and unique OTUs (197,743 and 13,849 respectively) while squamous cell carcinoma samples varied from 125,608 to 78,330 sequences and 3,659 to 2,924 OTUs respectively (Table 1).

### Beta and alpha diversity analyses

**Figure 1:**
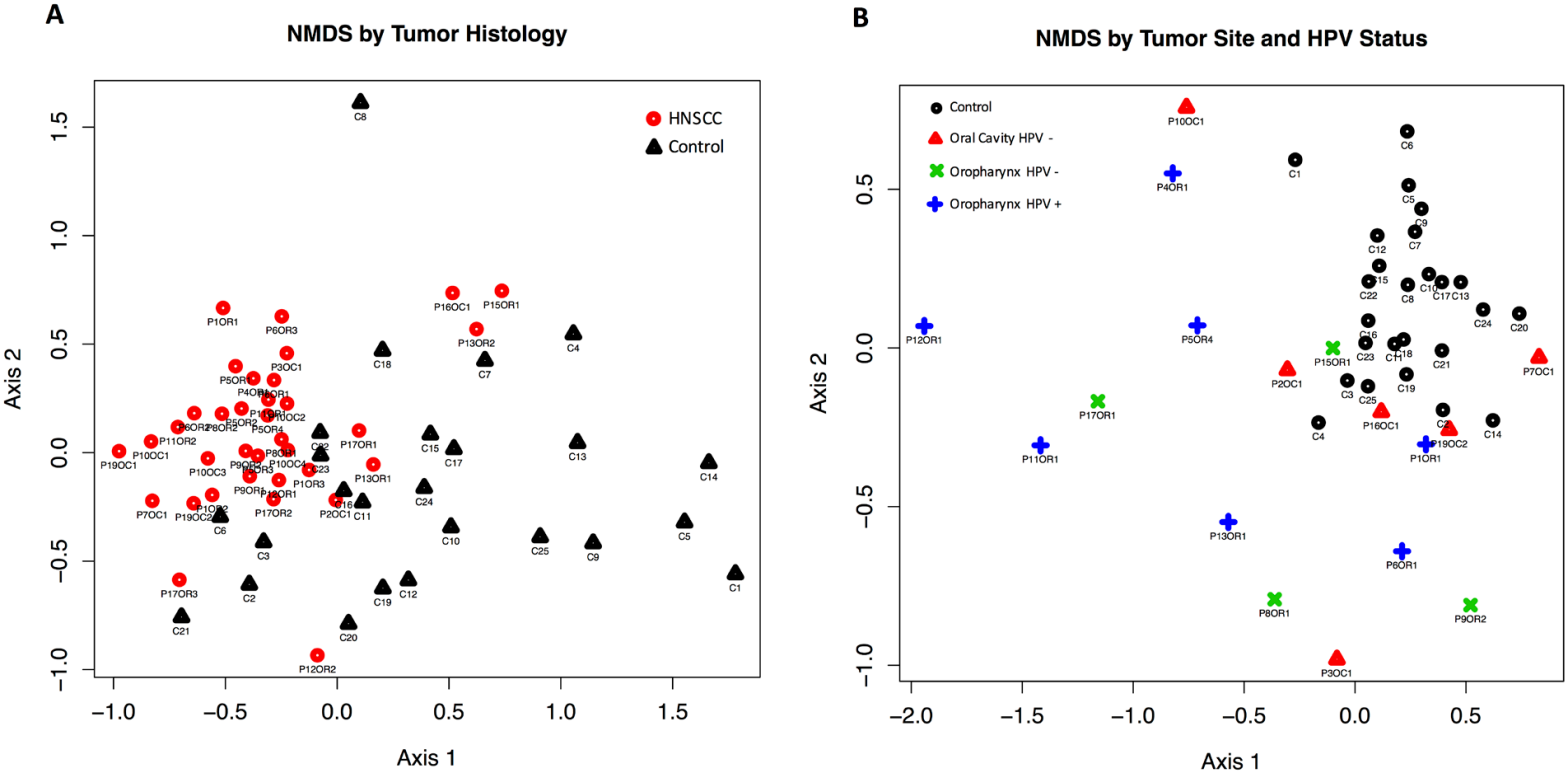
Beta diversity comparisons by Principal Component Analysis (PCA) using Non-metric multidimensional scaling (NMDS), with Euclidean distances, discriminated HNSCC (n=17) from Control samples (n=25) **A.** PCA reveals that the microbial communities in HNSCC patients are significantly different to those seen in Control samples. **B.** *NMDS* shows that the microbial communities in HPV negative (HPV-) oropharyngeal samples are significantly different from the ones seen in HPV-oral cavity patients.

Beta diversity comparisons using *non-metric multidimensional scaling (NMDS)*, discriminated HNSCC from normal samples (Figure 1, panels A and B). The 17 patient samples analyzed here corresponded only to the patient's first visit only. Microbial communities in oropharyngeal cancer samples were clearly separated from normal samples and oral cavity cancer samples. A slight discrimination can be observed also according to HPV status. Analyses of beta dispersion showed significantly higher variance in the OPSCC samples as compared to the controls and OSCC HPV negative samples (p= 0.02).

**Figure 2:**
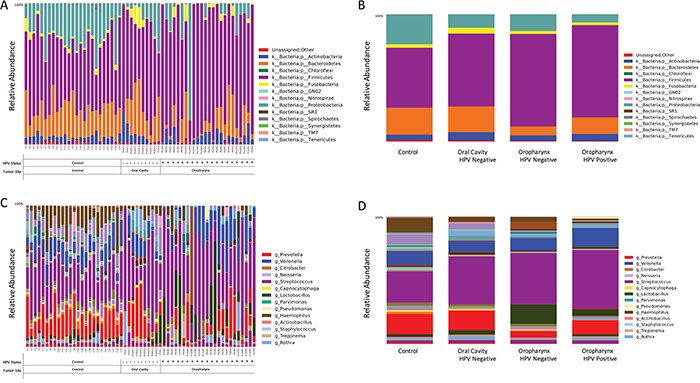
Taxonomic profiles at the phyla and genus levels, of 59 saliva samples according to tumor histology, HPV status and sampling site There were a total of 13 microbial communities identified at the phyla-level in our patient population **A.** The top 5 (>1% relative abundance) communities were, *Firmicutes, Bacteroidetes, Proteobacteria, Actinoabcteria* and *Fusobacteria* **B.** At the genus-level, *Streptococcus spp*. prevail across all samples, followed by *Veillonella, Prevotella, Lactobacillus* and *Haemophilus* spp **C-D.**

We found a total of 13 assigned phyla present, with 5 of these dominating across all of the samples: *Firmicutes, Proteobacteria, Bacteroidetes, Actinobacteria*, and *Fusobacteria*; with the other eight phyla having a relative abundance lower than 1%. In the control samples there was a dominance of *Firmicutes* (47.1%), *Bacteroidetes* (21.2%) and *Proteobacteria* (22.7%) while in the HNSCC samples the amount of *Bacteroidetes* (~13.4%) and *Proteobacteria* (~10.24%) decreased, and Firmicutes increased (~67%) (Figure 2A–2B). Overall, genus-level profiles showed a dominance of *Streptococcus and Prevotella* across all samples (Figure 2C–2D). *Lactobacillus* OTUs were more dominant in HNSCC samples (9.1%) compared to the controls (0.1%). *Neisseria* had higher abundance in oral cavity HPV negative samples (4.66%) compared to oropharynx (~1%) and significantly more abundant than in the controls, while *Citrobacter* was more dominant in OPSCC HPV negative samples (6.2%). *Haemophillus* OTUs were dominant in all HPV negative samples (~*4.1%*) as compared to HPV positive (1%). *Veillonella* OTUs were more abundant in OPSCC HPV positive samples (15%) as compared to HPV negative samples (9.4%) (Figure 2D). Tables representing the relative abundance values for each of the control and tumor samples for all patients at the phyla and genus levels can be found in the supplementary material section (Supplementary Tables 4 and 5, respectively).

**Figure 3:**
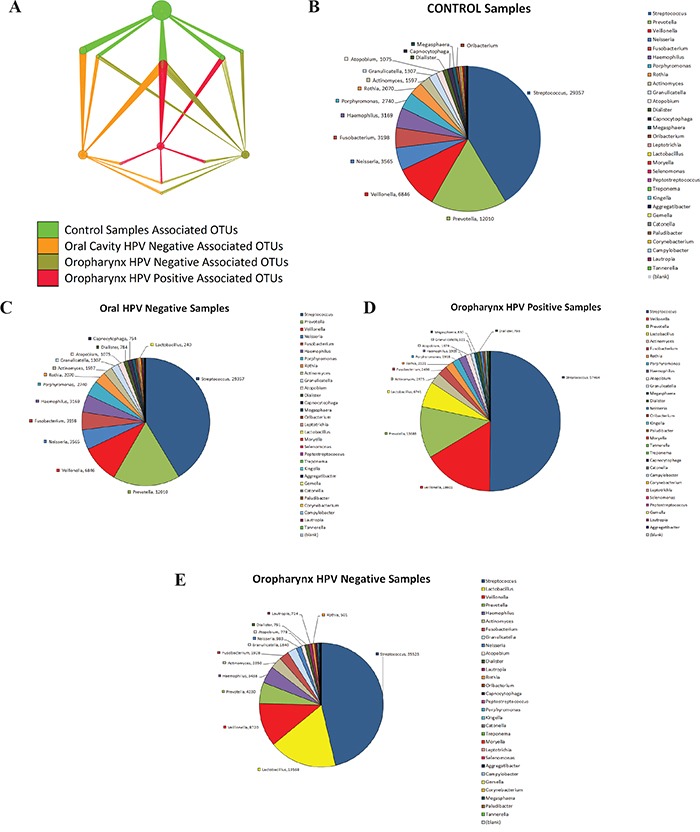
Nodes in Operational Taxonomic Units (OTUs) network significantly discriminate between head and neck squamous cell carcinoma (HNSCC) and normal samples in saliva The figure was created using QIIME and imported to Cytoscape. Significant OTUs p< 0.05 were plotted in the OTU Network. Pie charts were created showcasing taxa distinguishing samples by Tumor Histology and HPV status. **A.** represents taxa that differed significantly in relative abundance (p<0.05) when comparing saliva from normal patients with patients with HNSCC, as well as HPV negative and HPV positive patients. The OTU network shows that the total abundance of genus *Streptococcus, Dialister*, and *Veillonella* can be used to discriminate tumor samples from control samples **B.** Paired taxa within family Enterobacteriaceae together with genus *Oribacterium*, clearly distinguish OCSCC samples **C.** Paired taxa within family *Enterobacteriaceae* together with genus *Oribacterium*, clearly distinguish OCSCC samples (from OPSCC and normal samples **D.** and **E.**. Similarly, only HPV positive samples have an abundance of genus *Gemellaceae* and *Leuconostoc*.

The OTU network shown in Figure 3A represents taxa that differed significantly in relative abundance (p<0.05) when comparing saliva from normal patients with patients with HNSCC, as well as HPV negative and HPV positive patients (Supplementary Table 6). Overall, the network shows that the total abundance of genus *Streptococcus, Dialister*, and *Veillonella* can be used to discriminate tumor samples from control samples, which had close to half of their OTUs within genus *Haemophilus*, genus *Neisseria* and *Leptotrichia* (Figure 3B). Supplementary Figure 1A shows the significant difference in total abundance of *Veillonella* OTUs in HNSCC compared to normal. Supplementary Figure 1B shows the total abundance of significantly different *Veillonella* OTUs for each sample in the study, after subtraction of median total abundance of Veillonella OTUs from normal patients. Paired taxa within family *Enterobacteriaceae* together with genus *Oribacterium*, clearly distinguish OCSCC samples (Figure 3C), from OPSCC (Figure 3D and 3e) and normal samples. Similarly, only HPV positive samples have an abundance of genus *Gemellaceae* and *Leuconostoc* (Figure 3E).

**Figure 4:**
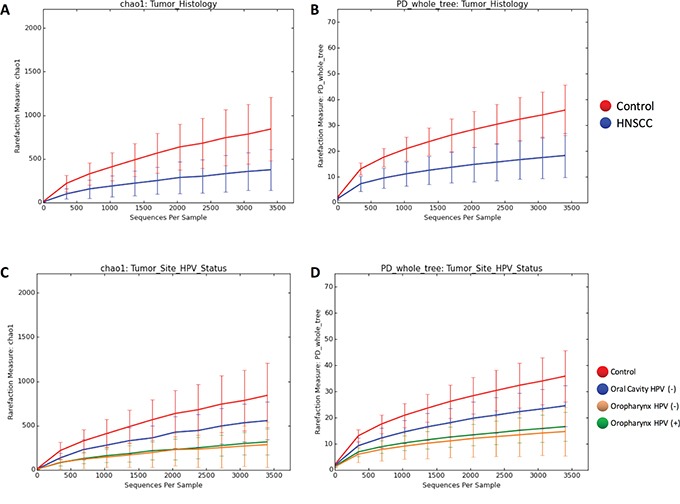
Rarefaction curves of species richness and diversity between Controls and HNSCC samples based on chao1 richness estimator and Faith's diversity measure (PD) Next-generation sequencing (NGS) has revealed a large microbial diversity that was previously concealed with culture-dependent methods. Although the true microbial diversity is limited to the number of samples, species richness and sample diversity can be estimated using diversity indices and species richness estimators. The chao1 index estimates total species richness based on all species actually discovered, including species not present in any sample. This approach uses the numbers of singletons (single appearance) and doubletons (that appeared twice) to estimate the number of missing species due to undetected species information is mostly concentrated on low frequency counts. Faith's phylogenetic diversity (PD) measure estimates the relative feature diversity of any nominated set of species by the sum of the lengths of all phylogenetic branches required to span a given set of taxa on the phylogenetic tree. **A.** shows the chao1 index estimates when comparing tumor versus control. **B.** shows the PD estimates when comparing tumor versus control. **C.** shows the chao1 index estimates when comparing tumor site and HPV status. **D.** shows the PD estimates when comparing tumor site and HPV status. Microbial communities of samples obtained from Control patients display significant higher richness (p=0.001, ANOVA) and significant higher diversity (p=0.001, ANOVA) than HNSCC samples. When considering HPV status and both sampling sites we found that the diversity from the oral cavity (HPV negative) HNSCC samples was higher than in both HPV positive (p=0.003, ANOVA) and negative (p=0.006, ANOVA) HNSCC samples from the oropharynx.

To better understand the OTU diversity in our cohorts, we compared the alpha rarefaction curves between normal and HNSCC sample categories according to the Chao 1 richness estimator and Faith's Phylogenetic Diversity index (see methods). Microbial communities from control samples display significantly higher richness (p<0.001) and significant higher diversity (p<0.001) than HNSCC samples (Figure 4A–4B). When considering HPV status and both sampling sites, we found that oral cavity (HPV negative) had a higher richness and diversity than oropharynx HPV positive (p<0.01) and negative (p<0.01) samples (Figure 4C–4D).

**Figure 5:**
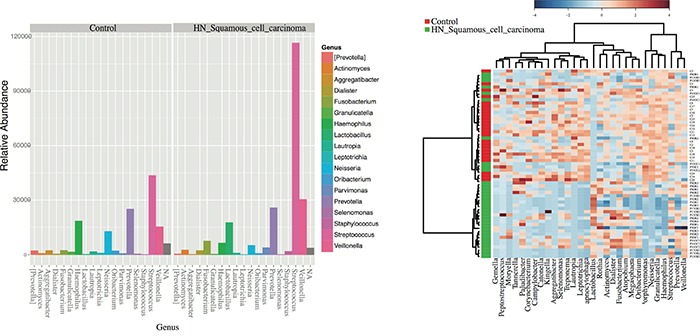
Differentially enriched microbiota OTUs in HNSCC when compared to control samples **A.** Histograms of the 42 statistically significant differences between OTUs abundance show a significant abundance of *Streptococcus spp*. in HNSCC samples according to G-tests results (p<0.05) By default, OTUs unclassified at the genus level are plotted as N/A by Phyloseq. **B.** Taxonomic Heatmap using Spearman's distance, combined with Ward clustering for 30 most statistically significant OTUs between Control and HNSCC samples (p<0.05, ANOVA). Heatmap color pallete used was “RdBu” with red and blue representing respectively low and high abundance. Ward's clustering method involves an agglomerative clustering algorithm that treats a cluster analysis as an analysis of variance, used to analyze the differences among group means and their associated classes. *Streptococcus and Lactobacillus spp*. are significantly associated with HNSCC samples according to G-tests and ANOVA results.

We also found significant differences in total abundance of *Streptococcus spp.* and *Lactobacillus spp*. OTUs in HNSCC samples when compared to control samples (p<0.05), using log-likelihood ratio tests and ANOVA in Qiime to compare the frequency of the OTUs. (Figure 5A) The taxonomic heatmap using Spearman's distance, combined with Ward clustering of statistically significant OTUs between control and tumor samples, revealed that *Streptococcus* and *Lactobacillus* spp. are significantly associated with HNSCC samples (p<0.05) (Figure 5B). The abundance of *Streptococcus*, *Peptostreptococcus and Tanerella* was significantly higher in the tumor samples compared to the controls. Control samples had significantly more abundance of *Agregatibacter, Lautropia, Haemophillus, Neisseria* and *Leptotricha*, taxa that were nearly lost in the tumor samples.

**Figure 6:**
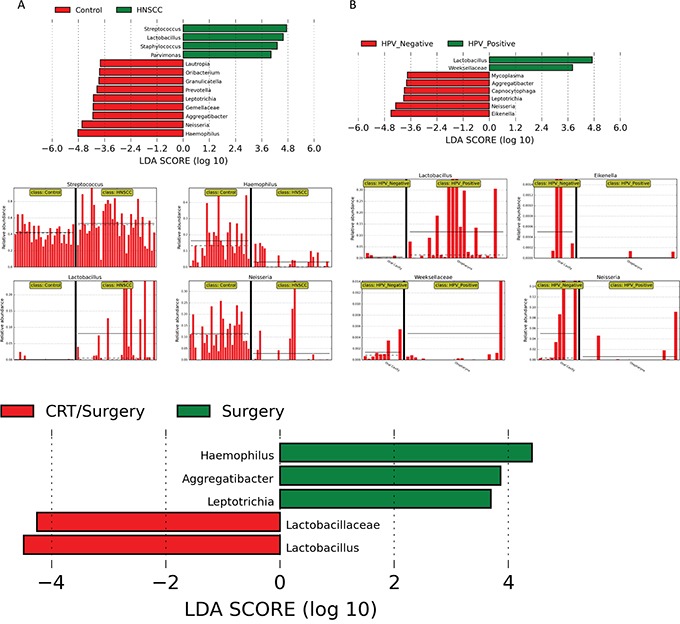
LDA Effect Size (LEfSe) algorithm was used on genus level OTU tables to determine taxa that best characterize each biological class: **A.** Comparison of HNSCC with normal samples found that *Streptococcus* and *Lactobacillus spp* are significantly associated with HNSCC samples. *Haemophilus* and *Neisseria spp*. are related to Control samples. **B.** Comparison of HPV positive and HPV negative HNSCC found that *Lactobacillus spp*. is significantly associated with HPV positive samples in the oropharynx. Similarly, *Eikenella* and *Neisseria spp*. are associated with HPV negative samples. Patients treated with surgery had significant enrichment for *Haemopilus, Neisseria, Aggregatibacter and Leptotrichia*, while patients treated with CRT/Surgery had significant enrichment for *Lactobacillus and Lactobacillaceae* **C.**

We used linear discriminant analysis (LDA) of effect size (LEfSe) to determine the taxa that most likely explains the differences between tumor and control samples. We compared taxa, not only on the basis of statistical significance, but also taking into account biological consistency of the results and effect relevance, to predict the best biomarker for each category. Significant differences were found between saliva microbiota from normal and HNSCC patients, as well as between HPV positive and HPV negative patients. These results confirmed the significant enrichment of *Lactobacillus* and *Streptococcus* in HNSCC, and additionally found an enrichment of *Staphyloccus* and *Parvimonas* compared to the controls (Figure 6A). We also found that *Haemopilus, Neisseria, Gemellaceae or Aggregatibacter* are more abundant in saliva from normal patients compared to HNSCC patients. Similarly, we found a profusion of *Lactobacillus* and *Weeksellaceae* OTUs in HPV positive samples, and an abundance of *Eikenella*, *Neisseria*, and *Leptotrichia* OTUs in HPV negative patients (Figure 6B). We also found significant pre-treatment differences in saliva microbiota from patients only treated with surgery, to patients treated with chemo-radiation therapy and surgery (CRT/Surgery). Patients treated with surgery had significant enrichment for *Haemopilus, Neisseria, Aggregatibacter and Leptotrichia*, while patients treated with CRT/Surgery had significant enrichment for *Lactobacillus and Lactobacillaceae* (Figure 6C).

### Longitudinal analyses of selected samples

We collected longitudinal post-treatment samples from a subset of 11 patients. We had two samples collected from six patients, three samples collected from three patients and four samples collected from four patients. The interval between sample collections ranged widely from 2 to 99 weeks, with a mean of 25.4 weeks, a median of 16.5 weeks and interquartile range of 25.4 weeks. Repeated samples were analyzed according to the type of treatment, HPV status and TNM staging based on relative abundance of OTUs.

**Figure 7:**
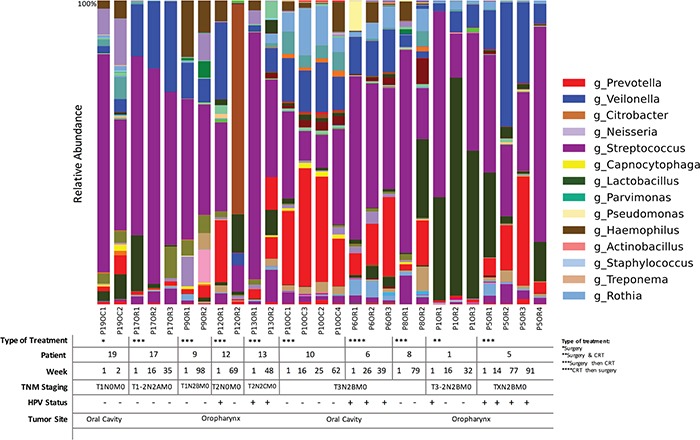
Time-series analyses of HNSCC patients (n=11) for whom we had repeated saliva samples, according to both sampling sites, HPV status and TNM staging Bacterial communities were noticeably different between T1-2N2AM0 and T3N2BM0 TNM stage. *Lactobacillus spp*. were significantly more abundant in patients with T3-2N2BM0 staging (p<0.01, *G*-test). Legend indicates genus that show a relative abundance higher than 1%.

We found that community structure fluctuated by patient, but not significantly across all patients. We did not observe significant longitudinal associations between community profiling and HPV status or TNM stage, probably due to small sample size and the wide range of intervals between repeated sampling. We did observe that while each patient had differentially abundant taxa between each time point, compared to each other overall, there was a decrease in *Streptococcus* as TNM stage progressed. Simultaneously, *Lactobacillus* OTUs increased in higher TNM stage categories across all patients (p<0.05). Although sample size is small to confirm any direct changes upon intervention, we found the increase in Lactobacillus post treatment to have occurred in 6 out of the 10 patients (patients # 19, 12, 13, 6 8 and 1). *Veillonella* increased in abundance after treatment in 73% of the patients. In fact, the patients with negative HPV results in salivary rinses, after initial HPV positive results before treatment, patients 1 and 12, had an increased dominance of *Lactobacillus* and a reduction in *Prevotella*. Overall fluctuations also occurred in the abundance of *Veillonella*, *Prevotela* and *Streptococcus*, when HPV status changed. A loss of HPV in the first patient resulted in a significant loss of *Lactobacillus* and increase in *Streptococcus* (Figure 7).

## DISCUSSION

The human ‘metagenome’ is a composite of Homo sapiens genes and genes present in the genomes of the trillions of microbes that colonize the adult body [25]. Genes from the latter are thought to outnumber the former by several orders of magnitude. The microbial genomes (the microbiome) encode metabolic capacities that we have not had to evolve wholly on our own, but remain largely unexplored [26, 27].

To our knowledge this is the first report that examines the saliva microbiome of OPSCC and the longitudinal changes observed in pre-treatment and post-treatment saliva microbiota. Several groups report high levels of colonization by microbiota in OSCC [12, 14, 28]. Microbiota colonization of OSCC tumors, bacterial translocation to the cervical lymph nodes and subsequent postoperative infection occurs in patients with oral cancer [29, 30]. Dozens of oral *Treponema* strains can be isolated from subgingival plaque samples found in patients with periodontitis [31]. *S. anginosus* thought to exist in the mouth as a normal flora, and to be located mainly in the gingiva and dental plaque, can also be located in the tumor, suggesting it is implicated in the carcinogenesis of head and neck squamous cell carcinoma [32]. More recently, viable bacteria, mostly saccharolytic and aciduric species [33], have been isolated from both superficial and deep tissue sections of OSCC [34], revealing that the tumor microenvironment is well suited for bacterial survival.

This study sheds light on the association of microorganisms with HNSCC cancer, which has not yet been adequately examined. We have shown that HNSCC cancer samples have decreased richness and diversity compared to controls although there are very few OTUs that change according to HPV status. Nonetheless, we found an interesting association of *Lactobacillus* OTUs with HNSCC cancer and HPV negative samples and a loss of *Prevotella* compared to the controls. This is the first report of *Lactobacillus* being significantly associated with HNSCC cancer samples, although these have been reported as major causes of caries, hyposalivation or xerostomia as well as in deep dentinal caries associated with pulpitis in adults [35]. We found an evident association of *Lactobacillus* OTUs with tumor samples. In patients from whom we had collected saliva samples at different time points, the abundance of *Lactobacillus* increased with advanced TNM stage. We also report that *Neisseria* is an abundant member of the oral cavity flora as it is significantly more abundant in HPV negative controls. Despite the fact that *Neisseria* are gram-negative taxa, certain OTUs seem to have been exploited for the design of vaccines comprising *N. lactamica*-derived antigens, *e.g.*, outer membrane vesicles (OMVs) [36, 37].

Significant differences in microbiota were also found when the linear discriminant analysis for effect size (LEfSe) algorithm was used on genus level OTU tables to determine taxa that best characterize each biological class, when comparing HNSCC from normal and HPV positive from HPV negative samples. LEfSe identifies features that are statistically different among biological classes (Kruskal-Wallis sum-rank test, p<0.05). It then performs additional tests to assess whether these differences are consistent with respect to expected biological behavior (Wilcoxon ran-sum test, p<0.05). Lastly, effect relevance is estimated by the linear discriminant analysis (LDA) effect size. LEfSe analysis confirmed the significant enrichment of *Lactobacillus* and *Streptococcus* in the HNSCC tumors found by OTU analysis with G-test and ANOVA, and additionally found an enrichment of *Staphylococcus* and *Parvimonas* compared to controls. In addition, we found that *Haemopilus, Neisseria, Gemellaceae* and *Aggregatibacter* are more abundant in controls, compared to the tumor samples. Similarly, we found an enrichment of certain *Lactobacillus* and *Weeksellaceae* in the HPV positive samples, and an abundance of *Eikenella*, *Neisseria*, and *Leptotrichia* in the HPV negative tumors used, when we used LefSe analyses to compare HPV positive and negative tumor samples.

Together this data suggests that the enriched presence of *Lactobacillus*, or the loss of *Haemopilus, Neisseria, Gemellaceae* or *Aggregatibacter* in saliva may be biomarkers of HNSCC cancer. We have also demonstrated that potential biomarkers for HPV+ HNSCC tumors include *Veillonella*, *Megasphaera* and *Anaerolineae*, which are anaerobic, saccharolytic and acid tolerant taxa. This is in agreement with the fact that the microenvironment of solid tumors is typically hypoxic with low pH, thus favoring the growth of this taxa [38].

The reduction of Proteobacteria (in our study taxa such as *Haemopilus or Neisseria)* in the tumors compared to the controls, was also found in a recent study where Proteobacteria taxa at all phylogenetic levels decreased in recent smokers while *Streptococcus* and *Atopobium* increased [39]. In fact, similar findings were reached in a study comparing the microbiota coating the tongue as potential source for diagnosing gastric cancer, and found that Proteobacteria also decreased in the oral microbiota of gastric cancer patients [40]. Changes in the oral microbiome in tumors, compared to controls suggest that the oral community structure may result in changes in functional pathways with systemic relevance.

Microorganisms cause an estimated 20% of human cancer [41, 42]. The best know example is the role *Helicobater pylori* plays in gastric cancer [43–45]. However, a handful of laboratories have reported links between bacterial infection and oral [11], colon [46–49], pancreatic [46, 50], liver [51], esophageal [52] and prostate cancers [53]. The biological mechanism of these associations is not yet understood [54, 55], however, there are several mechanisms by which bacterial infection could lead to the initiation and progression of oncogenic processes [42, 56, 57]. Bacterial endotoxins, metabolic byproducts of bacterial infection, and increased enzymatic activity as a result of bacterial infection, can induce somatic mutations and signaling pathway alterations [58]. Furthermore, the inflammatory cells and cytokines found in the tumor microenvironment of bacterial related chronic inflammation can lead to the creation of reactive oxygen and nitrogen species, which can also induce DNA alterations [24, 59, 60].

It has recently been shown that commensal bacteria can potentiate the effect of immunotherapy with check-point inhibitors. Oral administration of Bifidobacterium improved tumor control to the same degree as programmed cell death protein 1 ligand 1 (PD-L1)–specific antibody therapy, and combination treatment nearly abolished tumor outgrowth [61]. Similarly, the immunostimulatory impact of CTLA-4 blockade, leading to the antitumor effects, depends on distinct Bacteroides species. In mice and patients, T cell responses specific for B. thetaiotaomicron or B. fragilis are associated with the efficacy of CTLA-4 blockade [62].

We provide intriguing and preliminary data that evaluates the salivary microbiome and its relationship to head and neck cancer, HPV status, and its treatment. The major limitation of the study is the limited sample size. We are the first to show evidence of a repopulation or shift of the microbial communities seen in HPV positive HNSCC after surgical treatment. Our preliminary results suggest that the microbial diversity and taxonomic composition of the microbiota in saliva may be useful diagnostic and early detection biomonitors for HNSCC [63]. Potentiation of immunotherapy by the microbiota represents an important new concept to help explain the heterogeneity of anti-tumor immunity observed in the clinic. Although studies published today do not focus on microbiota specific to HNSCC, it seems very likely that the HNSCC microbiome could play similar roles as those shown in other tumors [64]. Further studies are needed to understand the etiologic role of the microbiome in HNSCC in general, and in OSCC and OPSCC specifically. Further studies are also needed to elucidate how specific microbiota affects immunotherapy. This is the first attempt at identifying taxa associated with HNSCC anatomic subsites, HPV status and surgical treatment status in pretreatment and post-treatment salivary rinses. Future work will determine the correlation of microbial communities in paired tissue and saliva HNSCC samples, as well as their link to treatment response and survival.

## MATERIALS AND METHODS

### Tissue and saliva

Tumor samples and salivary rinses from 19 HNSCC patients and 25 normal controls were obtained as previously described [65]. The oral rinses were performed with 20 mL of normal saline gargled twice, for 20 and 10 seconds respectively. Pre-operative tumor and saliva specimens were collected prior to commencement of any therapy at the Johns Hopkins Hospital. In cases where fresh frozen tumor DNA was not available, archival paraffin embedded tissue sections were obtained and *in situ* hybridization was performed as described previously [66], to assess the initial HPV-16 status. In total, we analyzed 19 cases with matched pairs of tumor and pre-treatment saliva DNA and 11 cases with tumor DNA, and both pre-treatment and from two to five post-treatment saliva DNA samples. Surveillance salivary rinses were obtained in the follow-up clinic visits from most of the HNSCC patients. Our protocol states that the first post-treatment rinse was collected 3 months after the completion of all treatment and then every 3 months for two years total. However, we were not able to obtain follow-up salivary rinses every three months for all patients so we included patients with at least one post-treatment salivary sample in this analysis.

### DNA extraction

Microdissected tissues and saliva (2mL) samples were centrifuged and the pellets were digested with 1% SDS and 50 μg/mL proteinase K (Boehringer, Mannheim, Germany) at 48°C overnight extracted with phenol/chloroform, precipitated in 100% ethanol, centrifuged at 5100 rpm for 45 minutes, washed in 70% ethanol twice, dissolved in LoTE buffer (10mM TRIS hydrochloride, 1mM EDTA buffer, pH 8), and stored at −20°C [67].

### Quantitative PCR

The 7900HT real time PCR system was used to perform quantitative PCR for HPV-16 *E6* and *E7* and *B*-actin. Specific primers and probes have been designed to amplify the *E6* and *E7* regions of HPV type 16: HPV-16 *E6* forward primer, 5′-TCAGGACCCACAGGAGCG-3′; HPV-16 *E6* reverse primer, 5′-CCTCACGTCGCAGTAACTGTTG-3′, HPV-16 *E6* TaqMan probe, 5′-(FAM)-CCCAGAAAGTTACCAC AGTTATGCACAGAGCT-(TAMRA)-3′, HPV-16 *E7* forward primer, 5′-CCGGACAGAGCCCATTACAA-3′, HPV-16 *E7* reverse primer, 5′-CGAATGTCTACG TGTGTGCTTTG-3′, HPV-16 *E7* TaqMan probe, 5′-(FAM)-CGCACAACCGAAGCGTAGAGTCACACT-(TAMRA)-3′. A housekeeping gene (*B-globin*) were run in parallel with HPV-16 *E6* and *E7* to standardize the input DNA: *B*-actin forward primer, 5′-TCACCCACACTGTGCCCATCTACGA-3′, *B*-actin reverse primer, 5′-CAGCGGAACCGCTCATTGCCAATGG-3′, *B*-actin TaqMan probe, 5′-(FAM)-ATGCCCTCCCCCATGCCA TCCTGCGT-(TAMRA)-3′. All samples were run in triplicate.

### HPV data analysis

The CaSki (American Type Culture Collection, Manassas, VA) cell line was used to develop standard curves for the HPV viral copy number as it is known to have 600 copies/genome equivalent. Standard curves for HPV-16 *E6* and *E7* were developed by using DNA extracted from CaSki cells, serially diluted into 50 ng, 5 ng, 0.5 ng, 0.05 ng, and 0.005 ng. A standard curve was also developed for the housekeeping gene *B-actin* (2 copies/genome), using the same serial dilutions of CaSki DNA. Tumor samples with ≥ 0.1 copy/genome and salivary samples with > 0 copy/genome were considered as HPV positive. Simple sensitivity and specificity analyses were performed on the cases with local recurrence. No statistical correlation was attempted due to modest sample size.

### Creation of the 16S rRNA V3-V5 amplicon library

An amplicon library from individual samples was created by PCR amplification with unique barcoded primers of the 16S rRNA V3-V5 gene region, using the 357F/926R primer set. We used 14 different barcode sequences and the linker primer sequence CCGTCAATTCMTTTRAGT to analyze the 16S rRNA V3–V5 hypervariable 16S rRNA gene region.

### DNA sequencing and bioinformatics analyses

Sequencing of the multiplexed amplified fragments was performed on the Roche/454 GS Junior pyrosequencing platform. Bioinformatics preprocessing steps included quality filtering, error-correction, and chimera removal. Briefly, reads were de-multiplexed using 5′ barcodes, trimmed of forward and reverse primer sequences, filtered for length and quality, and corrected for homopolymer errors. High quality reads were selected for analysis and reads with unknown bases (“N”) were discarded. The resulting high-quality dataset was then screened for chimeric sequences and contaminant chloroplast DNA.

Passing sequences were characterized for diversity and taxonomic composition using the Quantitative Insights into Microbial Ecology (QIIME) suite, version 1.9 where all the beta and alpha diversity measure and significance tests were performed [68, 69]. To begin, sequences were clustered into operational taxonomic units (OTUs) using UCLUST with a 97% identity threshold. Taxonomic assignment was performed using the RDP classifier (trained by a customized version of the comprehensive GreenGenes database, release v.13-05) with a minimum confidence threshold of 0.80. After considering the raw count data in full above, subsample analysis of each community was performed to an equivalent depth, in this case, 3,400 sequences per sample. All results are based on the subsampled data, which mitigates biases due to differences in sampling depth.

An OTU network was generated using QIIME [69] and imported to Cytoscape [70] based on OTUs that changed significantly in abundance (p<0.05) as result of a maximum likelihood statistical significance tests. The selected OTUs were plotted choosing nodes from the OTU network and sorting edges interaction by the four different sample types: normal, HPV negative OSCC, HPV negative OPSCC and HPV positive OPSCC. Additionally, we represented the taxonomy of taxa at the genus-level through pie charts at each of the four sample types.

### Statistical analysis

For each group comparison, significance tests were computed including the maximum likelihood statistical significance tests that determine whether OTU presence/absence is associated with a category in the metadata. The goodness of fit or log-likelihood ratio parametric test (*G*-test) compares the ratio of the observed OTU frequencies in the sample groups to the expected frequencies based on the null hypothesis (all sample groups have equal OTU frequencies). QIIME [69] was used to create all the heatmaps and estimate the following Alpha-diversity metrics: raw number of OTUs per sample, Chao1 estimator, Shannon entropy, Non-Metric dimensional scaling, and Bray-Curtis distance metric.

The chao 1 index approach for richness was used because it uses the numbers of singletons (OTUs with single appearance) and doubletons (OTUs that appeared twice) to estimate the number of missing species because missing species information is mostly concentrated on low frequency counts. Faith's phylogenetic diversity index (PD) estimates the relative feature diversity of any nominated set of species by the sum of the lengths of all phylogenetic branches required to span a given set of taxa on the phylogenetic tree. The relative group variance homogeneity was verified with the function ‘betadisper’ also in the “vegan” package. Richness box and whisker plots were calculated using both vegan [71] and Phyloseq [72] R packages.

We used linear discriminant analysis (LDA) with LefSe [73] an algorithm biomarker discovery that identifies taxa characterizing the differences between two metadata classes. It emphasizes statistical significance, biological consistency and effect relevance, allowing researchers to identify differentially abundant features that are also consistent with biologically meaningful categories (metadata), using non-parametric factorial Kruskal-Wallis (KW) sum-rank test, Wilcoxon rank-sum test and LDA. High LDA scores reflect significantly higher abundance of certain taxa.

## SUPPLEMENTARY MATERIALS














