# Supplementary material for: 16S rRNA amplicon sequencing identifies microbiota associated with oral cancer, human papilloma virus infection and surgical treatment
Source: Oncotarget. 2016 May 30;7(32):51320–34. doi: 10.18632/oncotarget.9710 (PMC5239478; doi:10.18632/oncotarget.9710)
Supplement: Supplementary file 1 [file oncotarget-07-51320-s001.pdf]

16S rRNA amplicon sequencing identifies microbiota associated with oral cancer, human papilloma virus infection and surgical treatment

SUPPLEMENTARY MATERIALS

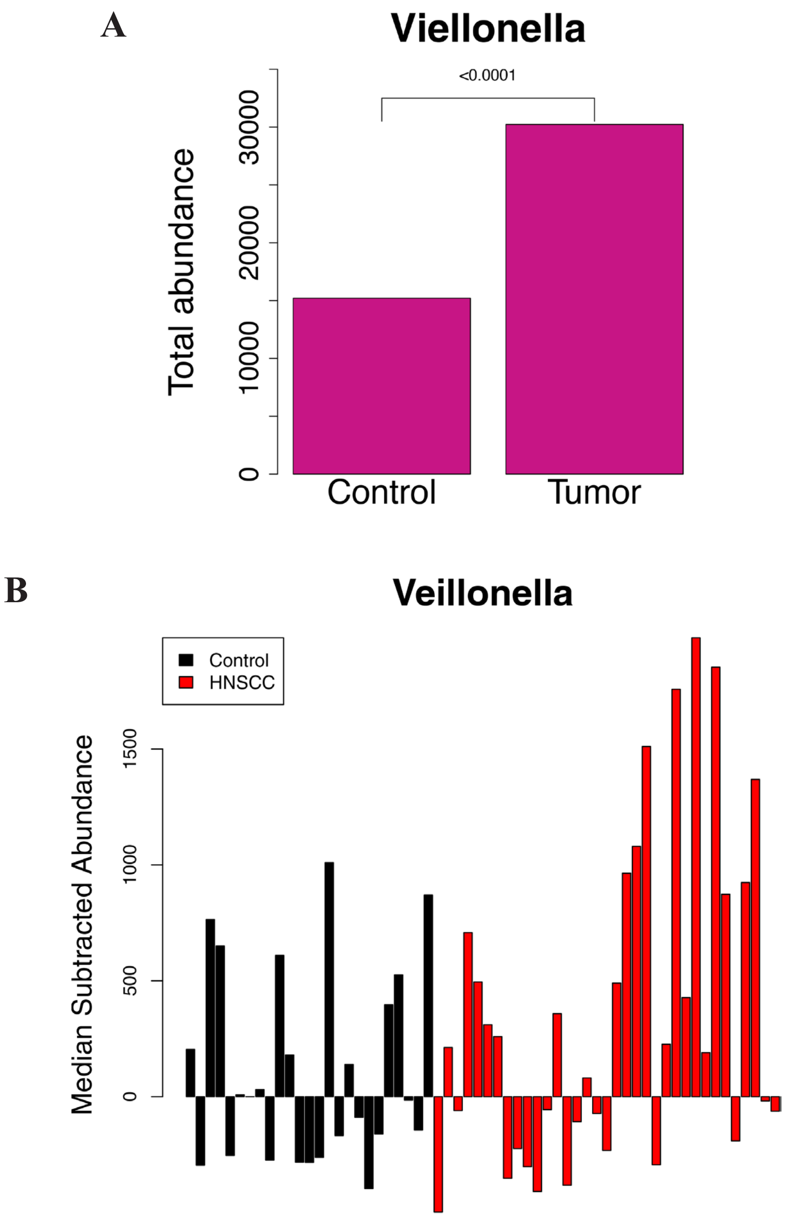

Supplementary Figure S1: Significantly differences in total and median subtracted abundance of Veillonella OTUs. A. Significant differences in total abundance of Veillonella OTUs in HNSCC compared to normal. **B.** Total abundance of significant Veillonella OTUs for each sample in the study, after subtraction of median total abundance of Veillonella OTUs of normal patients.

Supplementary Table S1: Head and neck cancer patients characteristics

See Supplementary File 1

Supplementary Table S2: Patient characteristics for repeated samples analysis

See Supplementary File 2

Supplementary Table S3: Barcode and linker primer sequences for 16S rRNA V3–V5 analysis

See Supplementary File 3

Supplementary Table S4: Detailed taxonomic string at the Phylum level with relative abundance for each of the studied samples

See Supplementary File 4

Supplementary Table S5: Detailed taxonomic string at the genus level, with relative abundance for each sample

See Supplementary File 5

Supplementary Table S6: Differential microbiota abundance in saliva from head and neck squamous cell carcinoma patients

See Supplementary File 6
